# Meningeal tuberculoma presenting as a space-occupying lesion in an immuno-competent young adult patient: A case report and literature review

**DOI:** 10.1016/j.radcr.2026.02.069

**Published:** 2026-03-30

**Authors:** Jeremia J. Pyuza, Furaha E. Kasyupa, Abitalis Mayengela, Agumbwike Mwakitwange, Deardiana Sengelela, Gilbert Nkya, Happiness Rabiel Massawe, Patrick Amsi, Angela Pallangyo, Alex Mremi

**Affiliations:** a Department of Pathology, KCMC University, 2240, Moshi, Tanzania; b Department of Pathology, Kilimanjaro Christian Medical Centre (KCMC), 3010, Moshi, Tanzania; c Kilimanjaro Clinical Research Institute, Moshi, Tanzania; d Department of General Surgery, Kilimanjaro Christian Medical Centre, Moshi(KCMC), 3010, Moshi, Tanzania; e Department of General Surgery, KCMC University, 3010, Moshi, Tanzania

**Keywords:** Meningeal tuberculoma, Space-occupying lesion, Imaging, Histopathology, Case report

## Abstract

Meningeal tuberculoma is a mass-like lesion formed by the immune response to *Mycobacterium tuberculosis*, involving the brain parenchyma, meninges, or both, that is characterized by central caseous necrosis surrounded by inflammatory cells and fibrosis. It is a rare but serious form of central nervous system (CNS) tuberculosis and can occur with or without tuberculous meningitis. We report a case of a 25-year-old female who presented with seizures and focal neurological deficits. Neuroimaging revealed a ring-enhancing lesion suggestive of a neoplastic process. The patient underwent left frontal craniotomy with gross total resection of the mass, and histopathological examination of the resected specimen confirmed caseating granulomatous inflammation consistent with tuberculoma, with Ziehl–Neelsen staining demonstrating acid-fast bacilli. The patient responded well to anti-tubercular therapy. This case highlights the importance of considering tuberculosis in the differential diagnosis of intracranial mass lesions, especially in endemic regions.

## Introduction

Tuberculosis (TB) remains a leading cause of morbidity and mortality globally, disproportionately affecting low- and middle-income countries (LMICs), where over 95% of TB-related deaths occur [1]. Although pulmonary TB is most common, extrapulmonary manifestations, including central nervous system (CNS) TB, account for up to 10-15% of cases in endemic settings [2], and account for close to 50% of all intracranial masses [3]. These parenchymal lesions may clinically and radiologically mimic primary brain tumors, particularly glioblastomas, due to their mass effect, ring enhancement, and associated midline shift [4]. This resemblance frequently leads to misdiagnosis, delayed treatment, and in some cases, unnecessary surgical intervention. Several reports have described patients undergoing craniotomy for presumed neoplasms before histopathology revealed tuberculous granulomas [5]. It mostly affects women aged less than 40 years.

These diagnostic challenges are exacerbated in LMICs by limited access to MRI interpretation expertise, stereotactic biopsy, and rapid TB diagnostics. Furthermore, sociocultural beliefs in many African settings contribute to delays in care. Neurological symptoms such as seizures, altered consciousness, or visual disturbances are often attributed to witchcraft, spiritual affliction, or adult-onset idiopathic epilepsy, resulting in poor health-seeking behavior and stigma [6]. The presence of comorbidities such as HIV infection and malnutrition further complicates diagnosis and worsens outcomes [7]. In this case, we report a young woman with imaging findings consistent with glioblastoma, who was ultimately diagnosed with cerebral tuberculoma following surgical resection and histopathology evaluation of biopsy, underscoring the need to maintain a high index of suspicion in radiological images especially in endemic regions.

## Case presentation

A 25-year-old female presented to a tertiary hospital with a 9-month history of progressive left-sided headache, recurrent episodes of loss of consciousness lasting approximately 1 minute, and focal tonic-clonic convulsions localized to the left side. Additional symptoms included blurred vision, dizziness, vomiting, and postural imbalance. She had initially sought care at a private hospital, where a CT scan revealed an intracranial mass, prompting referral to the tertiary hospital for further evaluation and management.

**Fig. 1:**
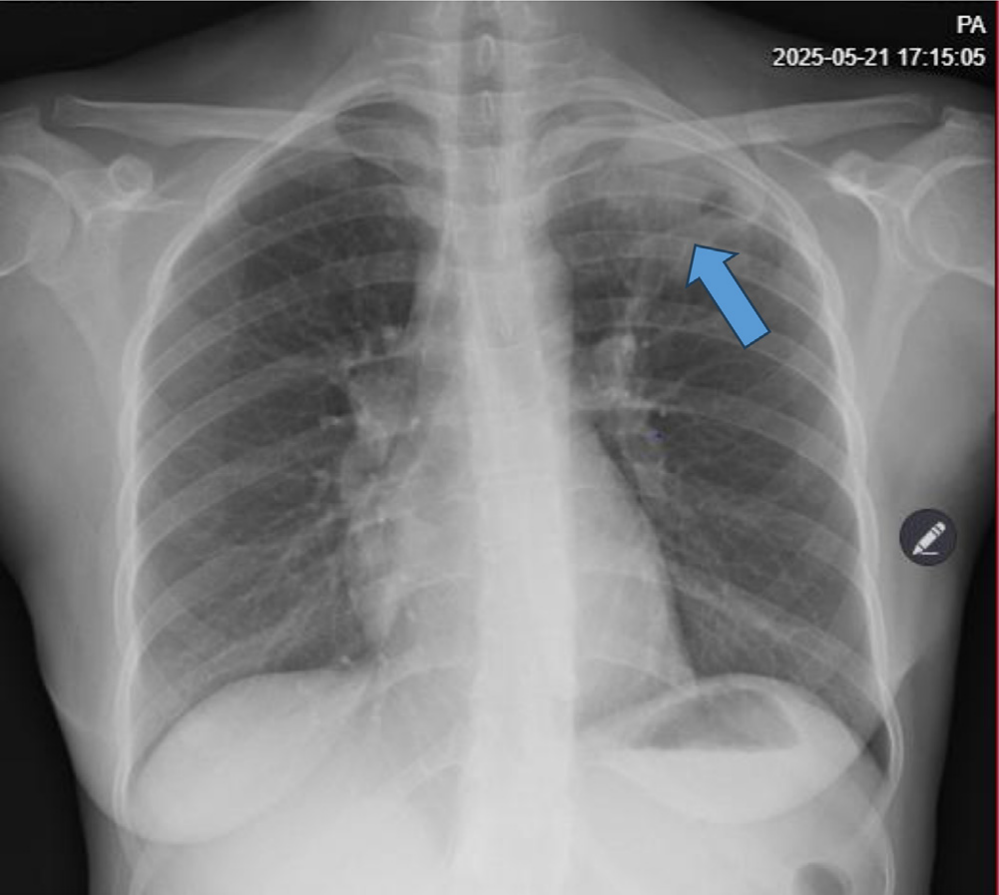
PA chest X-ray showing a homogeneous opacity in the left upper lung zone (blue arrow) with a suspicious cavitary component suggestive of pulmonary tuberculosis.

**Fig. 2:**
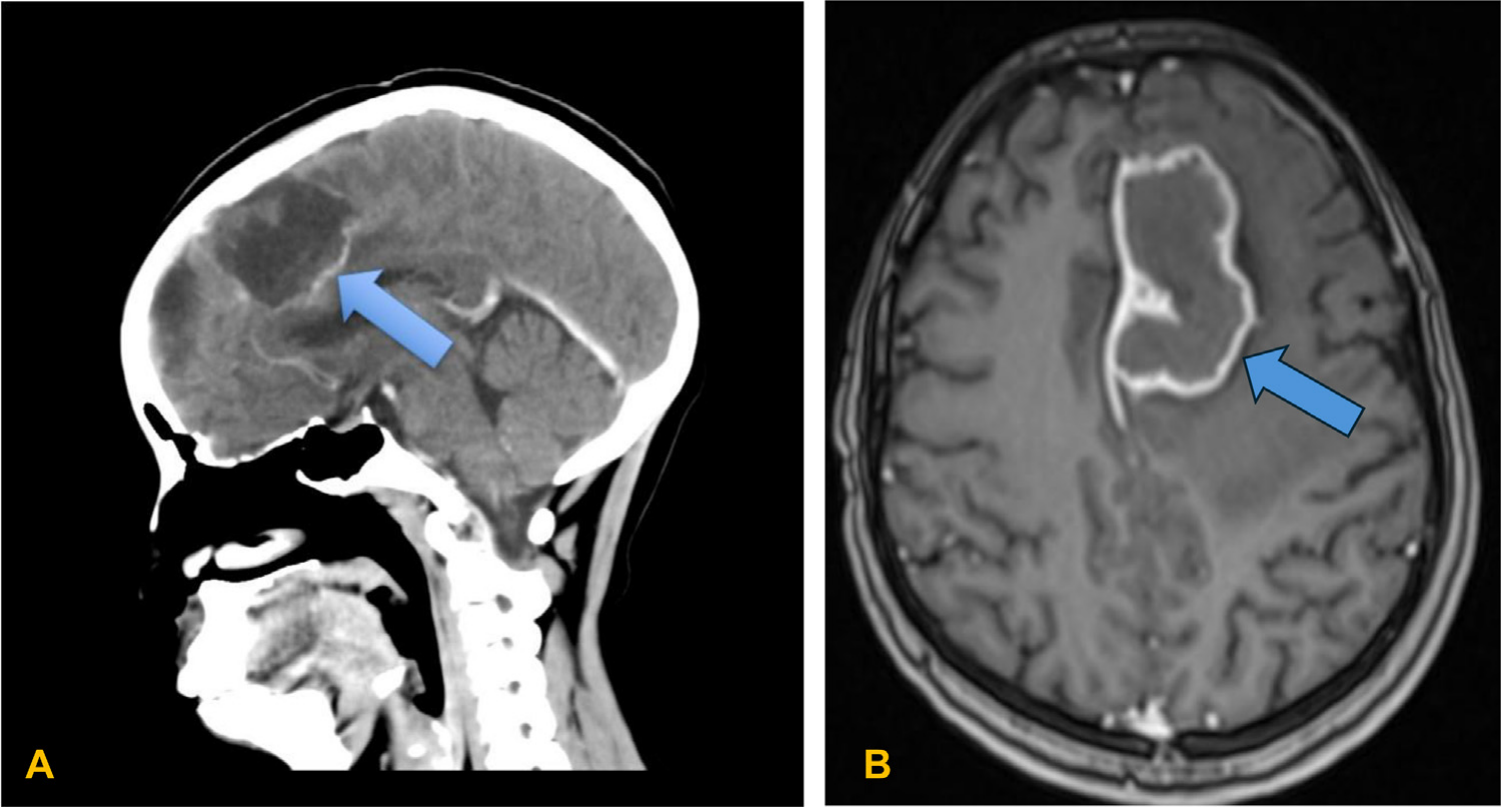
(A) Brain MRI showing a left parafalcine intra-axial mass (blue arrow). (B) Axial T1-weighted post-contrast image demonstrating a heterogeneous ring-enhancing anterior midline mass along the left parafalcine region, with mass effect on the ipsilateral frontal lobe, approximately 1.3 cm midline shift, and subfalcine herniation to the contralateral side.

Chest X-ray performed at the tertiary hospital showed a left upper lobe cavitary lesion suggestive of pulmonary tuberculosis (Fig. 1). Brain MRI demonstrated a left parafalcine intra-axial mass (Fig. 2A). Axial T1-weighted post-contrast imaging demonstrated a heterogeneous ring-enhancing anterior midline mass along the left parafalcine region with significant mass effect on the ipsilateral frontal lobe, approximately 1.3 cm midline shift, and subfalcine herniation (Fig. 2B).

**Fig. 3:**
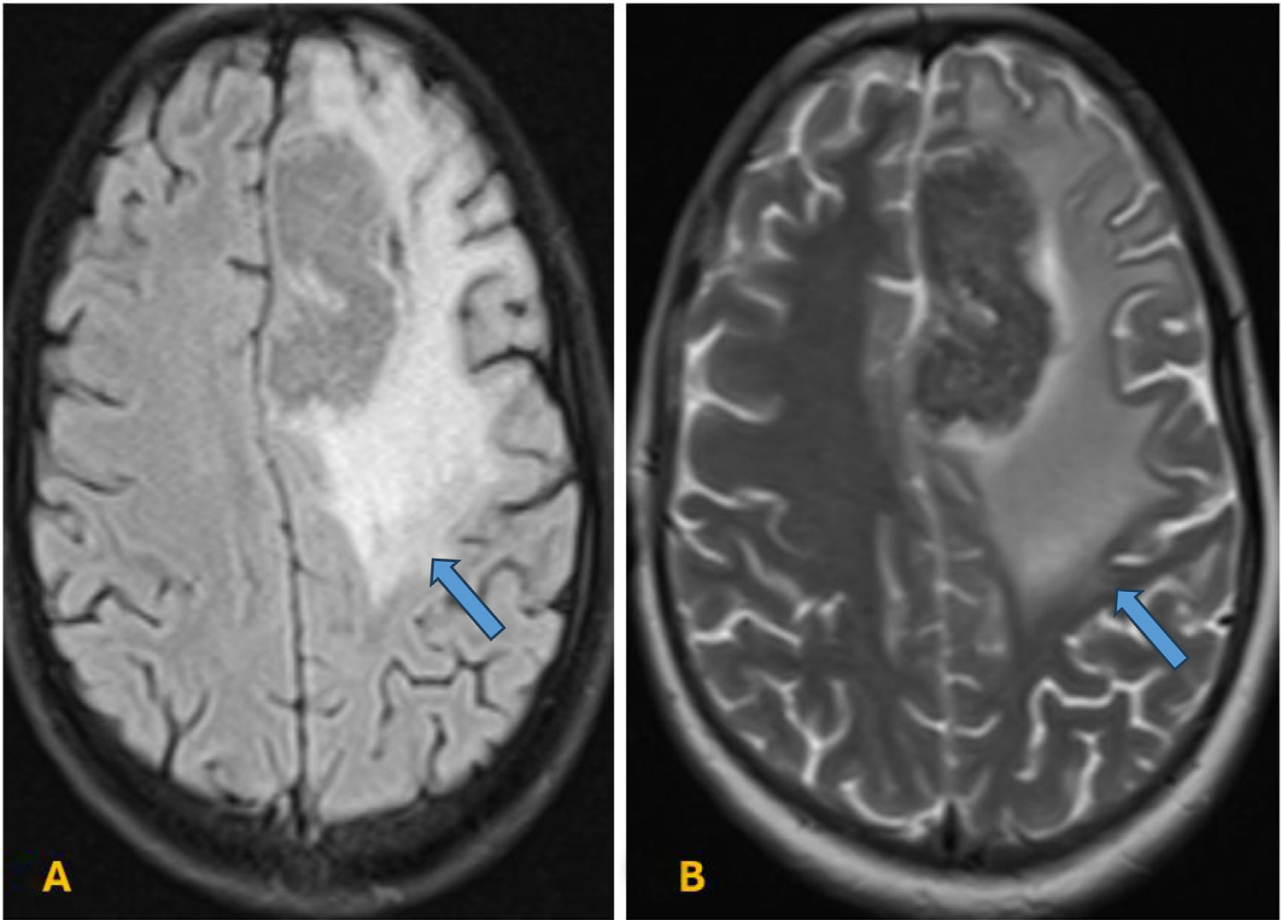
(A–B) Axial T2-weighted and FLAIR images highlighting extensive perilesional edema surrounding the lesion (blue arrow).

**Fig. 4:**
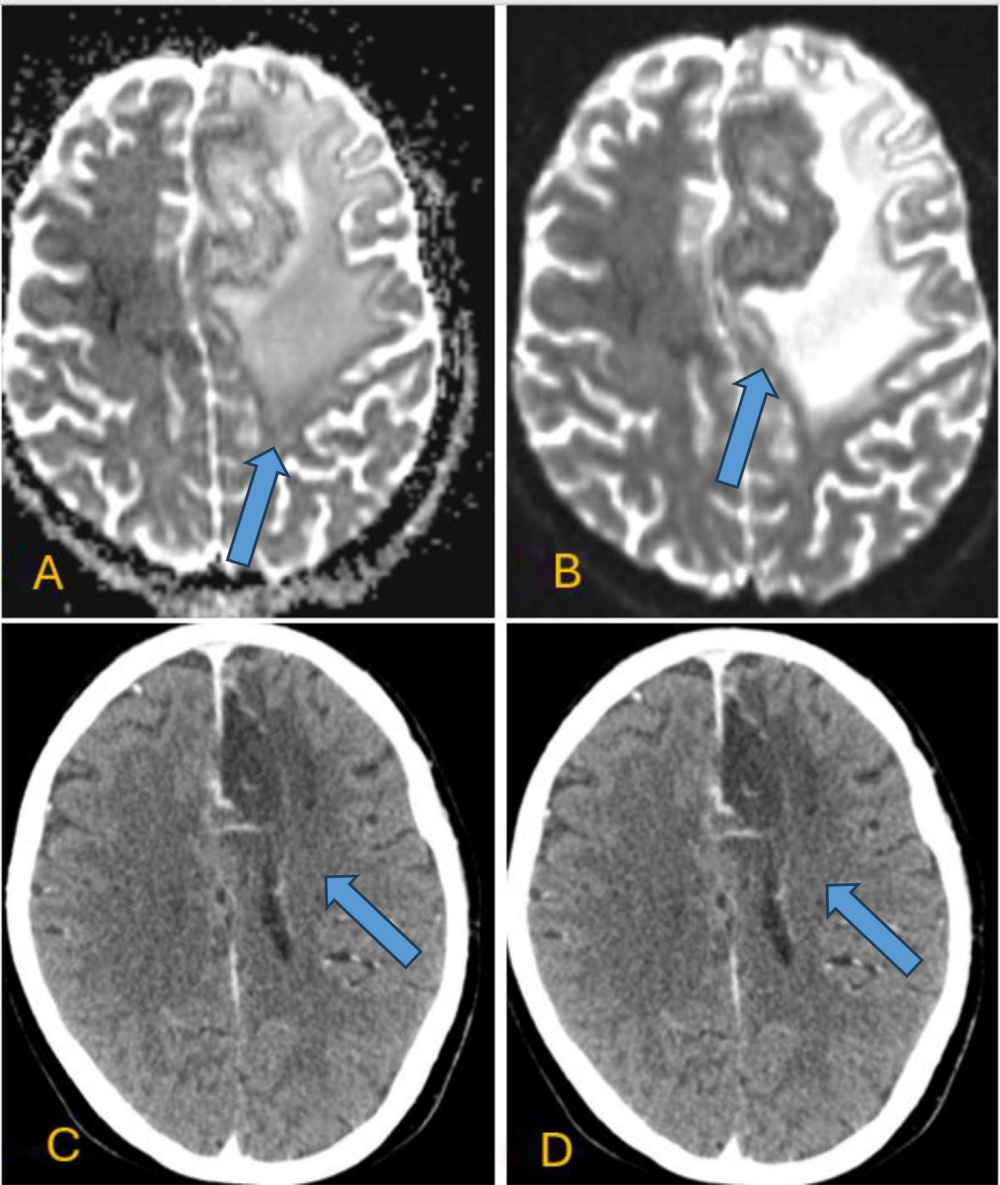
(A–B) Axial diffusion-weighted imaging (DWI) with corresponding ADC maps showing no diffusion restriction. (C–D) Axial plain CT scan (C) and axial post-contrast CT scan (D) of the brain following tumor resection, demonstrating a persistent ring-enhancing lesion in the left frontal lobe extending to the parafalcine region and crossing the midline. There is associated mass effect on the anterior horn of the left lateral ventricle, as well as the genu and body of the corpus callosum, with mild surrounding vasogenic edema.

Axial T2-weighted and FLAIR sequences highlighted extensive surrounding hyperintense vasogenic edema involving the left frontal lobe (Figs. 3A and B). Diffusion-weighted imaging (DWI) with corresponding ADC mapping showed no significant diffusion restriction within the lesion (Figs. 4A and B). The irregular peripheral enhancement, heterogeneous internal architecture, extensive vasogenic edema disproportionate to lesion size, and significant midline shift collectively contributed to the initial radiologic impression of high-grade glioma, particularly glioblastoma.

Laboratory investigations revealed leukocytosis (WBC 12.97 × 10⁹/L), normal hemoglobin levels (14.5 g/dL), platelet count of 374 × 10⁹/L), and normal renal function. Serology for HIV was negative. The patient had no history of chronic illness or immunosuppressive therapy and was therefore considered immunocompetent. Despite initial medical management, the patient continued to experience seizures, leading to neurosurgical planning for tumor resection. She underwent a left frontal craniotomy; intraoperatively, the lesion exhibited a necrotic appearance more typical of infectious pathology. Postoperative axial plain CT and axial post-contrast CT scans of the brain demonstrated a persistent ring-enhancing lesion in the left frontal lobe extending to the parafalcine region and crossing the midline, with associated mass effect on the anterior horn of the left lateral ventricle, genu, and body of the corpus callosum, with mild surrounding vasogenic oedema (Figs. 4C and D).

**Fig. 5:**
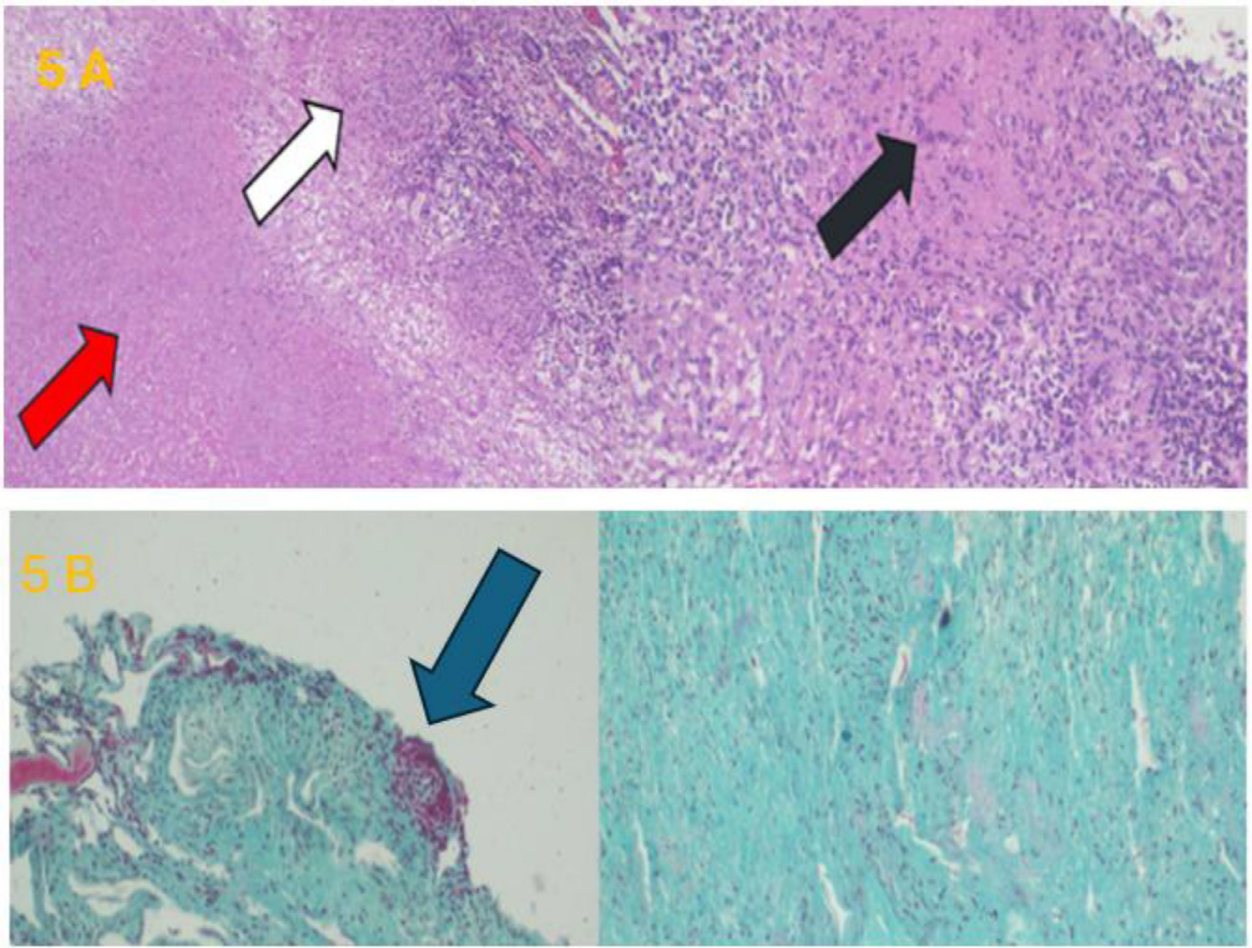
(A) Histopathological section showing central caseating necrosis (red arrow), epithelioid histiocytes (white arrow), and Langhans-type multinucleated giant cells (black arrow). (B) Ziehl–Neelsen stain demonstrating rod-shaped *Mycobacterium tuberculosis* acid-fast bacilli in red.

Histopathological examination of the resected brain tissue confirmed the diagnosis of cerebral tuberculoma. Microscopy showed central caseating necrosis (red arrow), epithelioid histiocytes (white arrow), and Langhans-type multinucleated giant cells (black arrow) consistent with tuberculous granulomatous inflammation (Fig. 5A). Ziehl–Neelsen staining demonstrated rod-shaped acid-fast bacilli in red (Fig. 5B). The final diagnosis was tuberculous brain granuloma (tuberculoma), initially misdiagnosed radiologically as glioblastoma. The patient was discharged on antiepileptic therapy and initiated anti-tuberculosis treatment, with subsequent clinical improvement.

## Discussion

Tuberculosis (TB) remains a major public health concern, especially in developing countries. Central nervous system (CNS) involvement is rare, accounting for ∼ 1% of TB cases, and can present as parenchymal or meningeal tuberculomas, often mimicking intracranial neoplasms [8,9]. Early diagnosis is crucial in preventing irreversible neurological damage. CNS tuberculomas result from hematogenous dissemination of *Mycobacterium tuberculosis*, either during primary infection or reactivation, seeding the leptomeninges or brain parenchyma [8]. Imaging is central to diagnosis, with contrast-enhanced MRI preferred due to superior sensitivity. Lesions may appear iso- or hypointense with a hyperintense rim on T2-weighted images in caseating tuberculomas [10]. However, radiological features vary with lesion stage, necrosis, and calcification.

In this case, a 25-year-old woman presented with progressive headache, seizures, and focal deficits. Brain MRI revealed a left parafalcine mass with midline shift, initially interpreted as glioblastoma. However, intraoperative and histological findings confirmed a tuberculous granuloma. This underscores the diagnostic difficulty in TB-endemic regions, where infectious lesions may mimic high-grade gliomas, particularly glioblastoma [10]. Glioblastomas typically show heterogeneous enhancement, necrosis, and perilesional edema, features that overlap with tuberculomas [[11], [12]]. The presence of a cavitary lung lesion on chest X-ray should have raised suspicion for TB dissemination. In retrospect, systematic integration of thoracic imaging findings with neuroimaging may have prompted earlier consideration of disseminated tuberculosis as the aetiology of the intracranial mass.

Ziehl–Neelsen staining may fail to reveal acid-fast bacilli in tuberculomas due to their paucibacillary nature. Thus, better radiological interpretation based on context and further confirmation using histopathology remains the diagnostic gold standard [13]. Misdiagnosis, as in this case, has therapeutic implications: glioblastomas often require resection, while tuberculomas respond to anti-TB therapy [14,15]. Here, surgery helped relieve the mass effect and obtain a definitive diagnosis.

Magnetic Resonance Spectroscopy (MRS), although not performed in this case due to technical limitations, may demonstrate lipid-lactate peaks suggestive of caseating necrosis in tuberculomas, whereas glioblastomas typically show elevated choline peaks with reduced N-acetylaspartate (NAA). Magnetization transfer ratio (MTR) imaging has also been reported to assist in differentiation, with granulomatous lesions demonstrating higher MTR values compared to infiltrative gliomas. Although cerebral tuberculoma is a recognized entity and larger case series exist in the literature, this report contributes by detailing a structured radiologic differentiation framework in an immunocompetent patient presenting with marked mass effect and herniation, features that strongly favored malignant pathology and created a clinically significant diagnostic pitfall. In future similar presentations, early integration of chest imaging findings, careful evaluation of T2 signal characteristics, consideration of advanced techniques such as MR spectroscopy or MTR imaging, and correlation with epidemiological context may help reduce diagnostic anchoring toward malignancy and improve clinical decision-making.

## Conclusion

This case highlights the diagnostic challenge of differentiating tuberculoma from glioblastoma on imaging. Given the reliance on neuroimaging in guiding management, accurate interpretation is critical to avoid unnecessary invasive procedures. In this instance, the patient’s favorable outcome following histopathological confirmation and subsequent anti-tuberculosis therapy underscores the importance of maintaining a high index of suspicion in TB-endemic settings. A structured approach integrating chest imaging, advanced MRI sequences, and clinical context may improve diagnostic accuracy in similar future cases, but the implementation is difficult in resource limited settings.

## Author contributions

Dr. Jeremia J. Pyuza prepared the case, followed up with the patient, collected data, and led manuscript drafting. Furaha Kasyupa participated in data collection and manuscript review. Dr. Deardiana Sengelela contributed to data collection. Dr. Abitalis Mayengela and Dr. Angumbwike Mwakitwange contributed to the manuscript review. Dr. Gilbert Nkya, Dr. Patrick Amsi, and Dr. Angela Pallangyo contributed to manuscript review, editing, and proofreading. Dr. Alex Mremi conceptualized the case report, provided overall supervision, and performed the final proofreading.

## Patient consent

Written informed consent was obtained from the patient. A copy is available for review upon request.

## References

[1] WHO. Global tuberculosis report 2023. 2023. [accessed 13.09.25]; Available from: https://www.who.int/teams/global-programme-on-tuberculosis-and-lung-health/tb-reports/global-tuberculosis-report-2023.

[2] Cherian A., Thomas S. (2011). Central nervous system tuberculosis. Afr Health Sci.

[3] Perez-Malagon C.D., Barrera-Rodriguez R., Lopez-Gonzalez M.A., Alva-Lopez L.F. (2021). Diagnostic and neurological overview of brain tuberculomas: a review of literature. Cureus.

[4] Mahamed Osman A., Abdullahi Karshe N. (2023). Atypical case of intracerebral tuberculoma mimicking glial tumor: a case report. Interdiscip Neurosurg.

[5] Shankar G.S., Nair S., Jacob T., Idikula M.J. (2023). Brain tuberculoma: a 52-year-old woman case report. Access Microbiol.

[6] Mushi D., Hunter E., Mtuya C., Mshana G., Aris E., Walker R. (2011). Social-cultural aspects of epilepsy in Kilimanjaro Region, Tanzania: knowledge and experience among patients and carers. Epilepsy Behav EB.

[7] Marais S., Pepper D.J., Marais B.J., Török M.E. (2010). HIV-associated tuberculous meningitis – diagnostic and therapeutic challenges. Tuberculosis.

[8] Rock R.B., Olin M., Baker C.A., Molitor T.W., Peterson P.K. (2008). Central nervous system tuberculosis: pathogenesis and clinical aspects. Clin Microbiol Rev.

[9] Thwaites G.E., Van Toorn R., Schoeman J. (2013). Tuberculous meningitis: more questions, still too few answers. Lancet Neurol.

[10] Bernaerts A., Vanhoenacker F.M., Parizel P.M., Van Goethem J.W.M., Van Altena R., Laridon A. (2003). Tuberculosis of the central nervous system: overview of neuroradiological findings. Eur Radiol.

[11] DeAngelis L.M. (2001 11). Brain tumors. N Engl J Med.

[12] McMahon P., Pisapia D.J., Schweitzer A.D., Heier L., Souweidane M.M., Roytman M. (2023). Central nervous system tuberculoma mimicking a brain tumor: a case report. Radiol Case Rep.

[13] Sahu C., Bhargava N., Singh V., Dwivedi P. (2020). Giant tuberculomas of brain: rare neoplastic mimic. J Pediatr Neurosci.

[14] Turan Suslu H., Bozbuga M., Bayindir C. (2010). Cerebral tuberculoma mimicking high grade glial tumor. Turk Neurosurg.

[15] Nahid P., Dorman S.E., Alipanah N., Barry P.M., Brozek J.L., Cattamanchi A. (2016). Official American Thoracic Society/Centers for Disease Control and Prevention/Infectious Diseases Society of America Clinical Practice Guidelines: treatment of drug-susceptible tuberculosis. Clin Infect Dis.

